# Tribological Behavior of Silver-Doped Diamond-like Carbon Coatings in Air and Simulated Biological Environments

**DOI:** 10.3390/ma19112349

**Published:** 2026-06-02

**Authors:** Łukasz Kołodziejczyk, Damian Batory, Anna Sobczyk-Guzenda, Agnieszka Maria Kołodziejczyk, Witold Szymański

**Affiliations:** 1 Institute of Materials Science and Engineering, Lodz University of Technology, Stefanowskiego 1/15, 90-537 Lodz, Poland; anna.sobczyk-guzenda@p.lodz.pl (A.S.-G.); witold.szymanski@p.lodz.pl (W.S.); 2 Department of Vehicles and Fundamentals of Machine Design, Lodz University of Technology, 1/15 Stefanowskiego St., 90-924 Lodz, Poland; damian.batory@p.lodz.pl; 3 Food Science Department, Faculty of Pharmacy, Medical University of Lodz, Muszynskiego 1, 90-151 Lodz, Poland; agnieszka.kolodziejczyk@umed.lodz.pl

**Keywords:** silver-doped diamond-like carbon coatings, tribological behavior, wear resistance, simulated body fluid, protein-containing medium, biotribology

## Abstract

**Highlights:**

- Ag-doped DLC coatings were systematically compared under identical sliding conditions in air, SBF, and protein-rich media.
- Increasing Ag content was associated with a lower H^3^/E^2^ ratio and increased coating wear.
- The friction response was governed mainly by the surrounding medium, with the lowest CoF observed in SBF.
- Surface analyses suggest that Ag affects protein-related surface features and transfer-layer evolution within wear tracks.

**Abstract:**

Silver-doped diamond-like carbon (Ag–DLC) coatings were investigated with respect to their tribological behavior under ambient and physiologically relevant conditions. Gradient Ag–DLC coatings deposited on AISI 316L stainless steel were tested in air, simulated body fluid (SBF), and an albumin-containing solution using a pin-on-disk configuration. Increasing silver content resulted in a systematic reduction in the H^3^/E^2^ ratio, leading to increased coating wear irrespective of the environment. In contrast, friction behavior was strongly controlled by the surrounding medium. Under dry sliding in air, all coatings exhibited similar steady-state friction governed by the DLC matrix. The lowest steady-state friction coefficients were obtained in SBF, indicating that the aqueous ionic environment provided the most favorable friction conditions among the tested media. In the albumin-containing medium, friction also remained low, indicating that protein adsorption and interfacial layer formation modified the sliding conditions, although the CoF did not fall below that observed in SBF. Wear was highest in air and generally lowest in SBF, while tests in albumin promoted surface layer formation. Surface analyses indicated silver redistribution, transfer-layer formation, and the presence of protein-related surface agglomerates, with higher apparent surface coverage on coatings containing more Ag. Overall, the results show that Ag-doped DLC coatings exhibit environment-dependent tribological behavior under physiologically relevant conditions. The present work should be regarded as a tribological study rather than a direct validation of antibacterial performance. Future studies should combine tribological assessment with dedicated antibacterial and cytocompatibility experiments.

## 1. Introduction

Amorphous hydrogenated carbon a-C:H coatings have become widely used materials mainly because of their excellent properties, such as their low coefficient of friction, high hardness, and good anti-wear and corrosion properties [1,2,3,4,5].

In addition to their typical application as low friction surfaces, DLC coatings are commonly used in implantology. The interest of the medical community in carbon coatings is due to their good biocompatibility in a tissue environment [5,6,7,8,9].

Diamond-like carbon coatings are well suited for lightly loaded applications, such as stents or intramedullary nails and body-piercing jewelry, due to their protection of the surrounding tissues against migration of metal ions released by the substrate (Ni, V, Co etc.) [10]. The application of DLC as a coating in artificial hip joints has aimed at lowering wear and preventing wear particle-induced osteolysis, which is the main reason for failed joint arthroplasty procedures [11]. Here, due to high loads, in addition to biocompatibility, the DLC layer must be characterized by elevated mechanical and tribological properties. An increasing number of studies have focused on improving biocompatibility and reducing bacterial cell adhesion by doping DLC coatings with elements that are toxic to microorganisms such as TiO_2_, Cu and Ag, just to mention a few. Recently, a great majority of studies have been devoted to carbon coatings doped with silver [4,12,13,14,15].

For a long time, silver has been known as an element that effectively fights many microorganisms [16,17]. The mechanism of bactericidal properties of silver is not fully understood; however, it is known that it is effective even in cases of microorganisms that have become resistant to a wide range of antibiotics [17,18].

The bactericidal properties associated with doping by silver should not, however, significantly affect the tribological properties of the base DLC coating. Manninen et al. studied DLC coatings doped with silver (1.3–13 at.%). They showed that for coatings containing silver up to 6.1 at.%, there was no significant deterioration in mechanical properties or wear resistance [14]. Thus, it appears that it is possible to achieve a compromise between antibacterial properties while maintaining the good mechanical and tribological properties (characteristic of pure DLC) of carbon coatings doped with Ag [15,19,20,21].

Hauert, in his review of the use of DLC in biomedical applications, concludes that the study of tribological properties of friction pairs working in the tissue environment should be conducted with the use of lubricants such as simulated body fluids [22]. There is evidence that the biomacromolecules present in biological fluids adsorb the articulating materials and strongly influence the mechanisms that determine their friction and wear behavior, as well as the type of wear particles produced [23,24,25,26,27].

Several studies have addressed the adsorption of proteins, like albumin and fibrinogen, to DLC in order to determine DLC’s hemocompatibility and appropriateness as a material for blood contacting implants (heart valves, blood pumps and stents) [28,29,30]. There is still disagreement on how the properties of the DLC surface affect protein adsorption, with some research suggesting hydrophilic surfaces are better for bearing surfaces and others suggesting hydrophobic surfaces are best [12,31,32,33,34]. The study by Carapeto et al. [35] focused specifically on the effect of albumin, the most abundant protein in periprosthetic fluid, on the tribological behavior of DLC/UHMWPE pairs.

Upon implantation, material surfaces are rapidly exposed to physiological fluids and become covered by a dynamically evolving protein layer. Competitive adsorption of proteins with different affinities to the surface occurs under physiological conditions, and both the composition and conformation of the adsorbed proteins play a decisive role in determining the biological and tribological response of the material. In particular, protein layers formed at the sliding interface can significantly modify friction and wear behavior by mediating contact conditions and influencing transfer and tribofilm formation [27].

Titanium and titanium alloys have been used in medicine for decades and remain among the most established metallic biomaterials for orthopedic, dental, and cardiovascular implants because of their favorable combination of specific strength, corrosion resistance, biocompatibility, and osseointegration ability [36,37]. Their application in components exposed to intensive articulation and wear is, however, more challenging, which has motivated extensive research on surface-engineering strategies aimed at improving tribological performance while preserving the advantages of the metallic substrate [5,36]. In this context, DLC coatings are particularly attractive because they can be deposited at relatively low temperatures by established vacuum-based techniques, including PVD- and CVD-derived methods such as PACVD/RF-PACVD and magnetron sputtering, which offer broad control over film structure, composition, and adhesion [5,38]. In parallel, additive manufacturing (3D printing) of titanium alloys has emerged as an important alternative route for producing patient-specific and porous implants; however, such components still often require additional surface modification to optimize their biological and tribological performance [37,39].

Recently, increasing attention has been devoted to multifunctional Ag-doped DLC coatings that simultaneously combine tribological performance with antibacterial activity. Contemporary studies emphasize that the main challenge is balancing antimicrobial efficiency with mechanical integrity and wear resistance, as higher Ag content typically reduces hardness while enhancing surface reactivity and biological functionality [15,19,20]. Furthermore, recent reviews highlight the significant role of surface chemistry and environment-dependent tribofilms in governing friction and wear in biomedical conditions, suggesting that protein-mediated lubrication and ionic environments must be considered in the design of DLC-based coatings [4].

Taking into account the above reports and the fact that, during the last few years, numerous reports related to the tribological behavior of DLC coatings in simulated body fluids have appeared in the scientific literature, the present work concerns the tribological properties of silver-incorporated diamond-like carbon coatings measured in simulated body fluid and bovine serum albumin. The use of an albumin-containing solution (most commonly serum albumin, e.g., bovine serum albumin, BSA) is justified by its ability to simulate the presence of proteins in body fluids and, therefore, to better reproduce the conditions under which an implant (biomaterial) functions in vivo. Following implantation, one of the first events is the almost immediate adsorption of plasma proteins, such as albumin, fibrinogen, and globulins, leading to the formation of a so-called conditioning film. As the major plasma protein (~40%), albumin rapidly adsorbs onto abiotic surfaces. Its presence simulates the initial biological interface and affects subsequent processes, including cell adhesion, inflammatory response, and overall biocompatibility [40]. This paper is a continuation of previous work related to the synthesis and characterization of silver-doped carbon coatings for external fixation devices, sensitive bacterial colonization and biofilm formation [15,27,41,42,43].

## 2. Materials and Methods

Samples for the investigation were made of commercial AISI316L (MEDGAL Orthopedic Implants & Instruments, Księżyno, Poland) austenitic steel. Chemical composition of the substrate material was examined by means of X-ray spectroscopy [43], giving the following surface composition (wt%): C 0.022, Si 0.583, Mn 1.669, P 0.021, S 0.022, Cr 16.48, Ni 13.38, Mo 2.49, Fe balance. The measured surface composition is consistent with the 316L stainless-steel family. Since the analysis was performed by a surface-sensitive X-ray method, these values represent surface composition rather than formal bulk chemical certification. It can be noted that compositional differences within the 316/316L family may influence ferrite content and related properties [44,45]. However, in the present work, all coatings were deposited and tested on substrates from the same material batch; therefore, the comparative tribological trends observed here are not influenced by substrate-to-substrate grade variation.

Cylindrical samples 16 mm in diameter and 6 mm thick were ground and polished using diamond suspension on a MECATECH 334 automatic polishing machine (PRESI, Eybens, France). Before loading into the working chamber, the samples were cleaned in ultrasonic cleaner in a methanol bath for 10 min. Gradient silver-incorporated carbon coatings were synthesized with the use of the hybrid Radio Frequency Plasma-Assisted Chemical Vapor Deposition/Magnetron Sputtering (RF PCVD/MS) method described elsewhere [43,46,47]. The process involves deposition of a Ti adhesion interlayer with a composition gradient (Ti → TiₓCᵧ → DLC) and a of thickness approx. 700 nm. Deposition was carried out at 10^−3^–10 Pa, 100–300 V bias, and a Ti target power of 40–80 W/cm^2^. DLC coatings were modified by silver magnetron sputtering in an Ar/CH_4_ atmosphere. Prior to deposition, Ar plasma etching was applied (1100 V, 2 Pa, 10 min). Deposition was performed at 600 V and 20 Pa for 3 min. Silver content was controlled by adjusting the magnetron power density in the range of 5–10 W/cm^2^.

Tribological parameters (friction coefficient and wear rate) were determined using the pin-on-disk method. Pin-on-disk tests were performed using a CSM tribometer (CSM Instruments, Peseux, Switzerland) under 1 N with a sliding speed of 0.1 m/s for approx. 315 m in air and simulated physiological solutions. A ¼-inch-diameter commercial AISI 316L medical-grade steel ball was used as the counterpart. According to the manufacturer’s specification, the steel balls were produced in accordance with ISO 3290 [48], class G10, corresponding to a surface roughness of Ra = 0.025 µm. The surface roughness of the coatings was evaluated by atomic force microscopy, yielding RMS roughness values of 1.67 nm for Ag4, 2.78 nm for Ag8, and 3.36 nm for Ag15 [29]. Pin-on-disk tests were carried out under dry friction conditions in ambient air at 24 °C and 30–40% relative humidity.

Additional tribological tests under lubricated conditions were performed at 37 °C in two media: simulated body fluid (SBF) and simulated body fluid containing bovine serum albumin. In the following, the albumin-containing medium is referred to as BSA, as the tribological response is dominated by protein adsorption rather than by inorganic ions. Prior to the tests in BSA, the samples were incubated in the solution for 1 h at 37 °C to allow for protein adsorption. Table 1 shows the appropriate amounts of reagents added to deionized water (diH2O) for the preparation of SBF. SBF was adjusted to the desired pH7.4 using 1 M HCl. The concentration of albumin in the simulated body fluid was 50 g/L (concentration of albumin in human blood plasma). Each tribological condition was tested in three independent experiments. The reported values are given as mean ± standard deviation.

After the tribological tests, the resulting wear tracks and their profiles were analyzed with a cross-profiling option on a G200 Nano Indenter (KLA-Tencor Corporation, Milpitas, CA, USA) equipped with a conical diamond probe tip with an apex angle of 90 deg and 1 μm radius. MountainsMap Premium 5 (Digital Surf, Besançon, France) software was used to calculate the area of the wear-track cross-section profiles. Finally, based on the cross-section areas, the specific wear rate for each sample was determined.

For observation of the wear tracks after the tribological tests, an optical microscope MA 200 Eclipse (Nikon, Tokyo, Japan) with a digital data acquisition system was used. Measurement of characteristic dimensions of the disk and the counterpart after the test (width of the wear track on the disk and ball wear diameter) was performed using NIS-Elements Basic Research Software 3.2 (Nikon, Tokyo, Japan). These measurements were used for qualitative comparison of the contact geometry, while the quantitative wear rates were determined based on profilometric analysis. Chemical composition of the wear track and transfer layer formed on the counterpart were examined using an S-300N scanning electron microscope (Hitachi, Tokyo, Japan), equipped with a Thermo Noran EDS analyzer (Thermo Fisher Scientific, Waltham, MA, USA).

Surface morphology and topography were measured using a BioScope Resolve atomic force microscope equipped with a Nanoscope V controller (Bruker Corporation, Billerica, MA, USA). All investigations were performed under ambient conditions. Topography measurements were made in tapping mode, and the size of the images was 1 × 1 µm^2^ and 10 × 10 µm^2^. Commercial silicon HQ:CSC15 (MicroMasch, Tallinn, Estonia) cantilevers with a nominal tip radius of ~8 nm and nominal cantilever spring constant of 0.3–0.8 N/m were used. Image acquisition was performed using Nanoscope 9 software, and further image processing was done using Nanoscope Analysis 1.9 (Bruker Corporation, Billerica, MA, USA) and SPIP 6.4 (Image Metrology A/S, Kongens Lyngby, Denmark) software. Additionally, from the topography images of the samples tested in BSA solution, the albumin surface coverage parameter (SC) and the average size of the agglomerates were defined (calculated for 10 × 10 µm^2^ scan size images). The prepared topography images were processed using the Pore and Particle analysis option in Watershed–Dispersed Features segmentation mode (this method is primarily used for detecting dispersed particles and/or pores on a background that are too wavy for the classic threshold methods or when the distribution of heights/depths is too wide).

The chemical structure of the coatings was characterized prior to tribological testing and discussed in detail in our previous work [29]. Therefore, post-test Raman analysis was not included in the present study. In the absence of Raman spectra from the wear tracks, graphitization-related interpretations in this manuscript are treated as indirect and literature-consistent rather than directly proven.

Fourier Transform Infrared Spectroscopy (FTIR) analysis was conducted using a Nicolet iS50 spectrometer (Thermo Scientific, Waltham, MA, USA) with a measurement range from 4000 cm^−1^ to 400 cm^−1^ and a resolution of 0.5 cm^−1^. A DTGS KBr beam splitter was used. Measurement was performed in absorbance mode, using the diffuse reflectance DRIFT accessory (Harrick Scientific Products, Pleasantville, NY, USA), at a beam angle of incidence equal to 30°. The number of scans per measurement cycle was 120.

## 3. Results and Discussion

### 3.1. Characterization of Ag-Doped DLC Coatings

In our previous paper [43], the main characterization of the physiochemical and mechanical properties of Ag-doped DLC coatings was presented. Three different silver concentrations in synthesized coatings were obtained (4.5, 8.4 and 15.2 at.%). Later, in this work, samples with varied Ag surface content are defined as Ag4, Ag8 and Ag15, respectively. XPS investigation revealed that silver incorporated in an amorphous diamond-like carbon matrix occurs mostly in the metallic state, without bonding to the carbon atoms [50,51]. However, the research provides no information about the size and distribution of the deposits in the matrix [50]. The SEM study showed randomly distributed silver conglomerates on the surface as well as its partial dissolution in the amorphous carbon matrix.

The mechanical property results (hardness and Young’s modulus) reported in our previous work [43] allowed calculation of the H^3^/E^2^ ratio linked to the plasticity index, what is presented in Table 2.

The H^3^/E^2^ term combines the H and E values of a material and sets the amount of elasticity exhibited by the film [52,53]. In particular, high values of H^3^/E^2^ mean that a highly elastic behavior of the film under contact events is achieved. The H^3^/E^2^ parameter is widely used as an indicator related to the elastic–plastic response of the coating under contact loading [53]. In the present system, the decrease in H^3^/E^2^ with increasing Ag content is consistent with the observed increase in coating wear. However, the tribological response is also influenced by the testing environment and by interfacial processes occurring during sliding; therefore, H^3^/E^2^ should be interpreted as an important contributing parameter rather than as the sole determinant of wear behavior. The scratch test results of the synthesized coatings showed that the incorporation of silver results in a reduction in the critical delamination force [43]. Nevertheless, the mechanical properties of the examined coatings are still maintained at reasonably high levels.

A similar relationship between reduced H^3^/E^2^ values and increased wear has been widely reported for non-carbide-forming metal dopants, where silver acts primarily as a surface-active modifier rather than as a reinforcing phase. In such systems, the deterioration in wear resistance is considered a tradeoff between mechanical compliance and functional surface modification, rather than an indication of coating failure [54,55].

### 3.2. Coefficient of Friction

**Figure 1:**
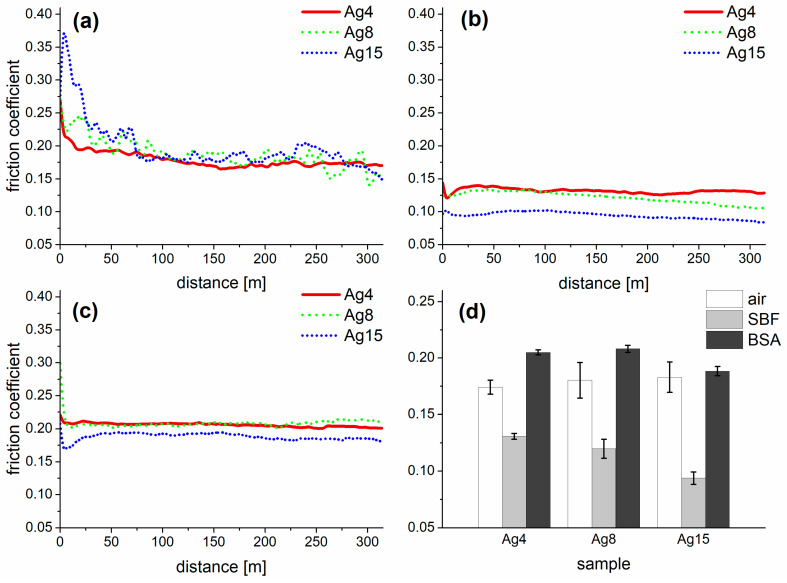
Evolution of the coefficient of friction (CoF) recorded during pin-on-disk tests of Ag-DLC coatings performed in (**a**) air, (**b**) simulated body fluid (SBF), and (**c**) bovine serum albumin solution (BSA). Panel (**d**) shows the average steady-state CoF values calculated from the stable sliding regime.

In Figure 1a, the evolution of the friction coefficient during pin-on-disk tests in ambient air for the investigated samples is presented. All the measured samples show similar friction curves in the steady-state regime (which follows an initial running-in period of approximately 50 m); however, Ag4 exhibited slightly smoother friction curves throughout the test than DLC doped with higher concentrations of Ag. Moreover, for all Ag-DLC samples, the friction curves start at relatively high values in the running-in period (approx. 0.26–0.36), and after a gradual decrease, they converge to a steady state (approx. 0.18), indicating that under dry sliding conditions, the friction response is governed primarily by the DLC-rich matrix rather than by the silver content.

CoF evolutions for the pin-on-disk tests performed in physiological solutions are depicted in Figure 1b,c. For both SBF and BSA media, the steady-state sliding period is characterized by much smoother curves than in the ambient atmosphere (Figure 1a). For comparative purposes, the average steady-state friction coefficients calculated from the stable sliding regime are summarized in Figure 1d. The reported coefficient-of-friction values correspond to the average calculated over the steady-state portion of the friction curve, excluding the running-in stage. While the full friction curves provide insight into the running-in behavior and stability of the contact, the averaged values allow a direct comparison of the steady-state tribological performance of the investigated coatings under different environments. The steady-state friction reflects contact conditions dominated by the deeper DLC-rich regions of the coatings. According to Figure 1d, the lowest steady-state CoF values are obtained in SBF for all investigated coatings. This indicates that the aqueous ionic environment provides the most favorable friction conditions among the tested media, most likely by reducing direct solid–solid interaction at the sliding interface. In contrast, dry sliding in air leads to slightly higher CoF values, although the differences between coatings remain small. The albumin-containing medium also provides relatively low friction; however, the CoF values do not decrease below those observed in SBF.

The friction behavior in BSA suggests that protein adsorption modifies interfacial shear conditions and contributes to the formation of a surface layer during sliding. At the same time, the absence of a further friction reduction relative to SBF indicates that, under the present test conditions, protein-mediated boundary lubrication does not surpass the friction-lowering effect associated with the aqueous ionic medium alone. Therefore, the results demonstrate that the friction response of Ag–DLC coatings is controlled mainly by the test environment, whereas the influence of Ag content on steady-state CoF remains secondary. Environment-dependent friction behavior has also been reported for carbon-based coatings tested in aqueous and protein-containing media, where variations in lubrication conditions and interfacial interactions are likely to influence the macroscopic friction response [55,56].

### 3.3. Wear Rate

**Figure 2:**
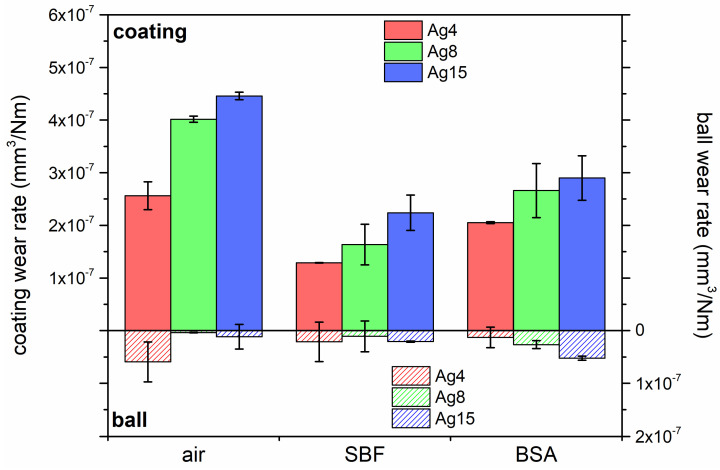
Specific wear rates of Ag-DLC coatings and AISI 316L steel counterparts determined after pin-on-disk tests conducted under a normal load of 1 N and a sliding distance of 315 m in air, SBF, and BSA. Coating wear is shown by solid bars, while counterpart wear is indicated by patterned bars.

Many tribological studies focus primarily on coating wear, neglecting the response of the counterpart, which represents an equally important part of the tribological system. For real application use, the lack of data for the counterparts is less than useful, as reducing any wear debris is crucial for both contacts [57]. In Figure 2, the specific wear rate for the coatings and counterparts after the pin-on-disk tests performed in all three media is presented (Hertzian contact pressure and contact radius are approx. 520 MPa and 30 µm, respectively). It is clearly visible that, regardless of the test environment, an increase in Ag surface concentration leads to an increase in disk wear rate. For all types of Ag-doped DLC coatings, the smallest wear is observed in the SBF environment, while the highest wear is noticed for tests in air. With respect to counterpart wear, the highest value (7.6 · 10^−8^ mm^3^/Nm) was observed for DLC doped with the lowest Ag content in air.

The only wear dependence on Ag content was registered for samples tested in BSA solution, where a gradual wear increase with Ag concentration increase is observed. Nevertheless, the wear in the counterparts is very small compared to the wear of these coatings.

Although clear trends in coating wear can be observed, particularly with increasing Ag content, the wear rates of the steel counterparts remain very low in all tested environments. Therefore, the differences observed for the counterpart wear should be interpreted with caution, as they may partially fall within the experimental scatter inherent to low-wear tribological systems.

The lack of a direct correlation between steady-state friction coefficients and wear rates is consistent with previous studies on Ag-doped DLC systems, indicating that silver primarily influences surface chemistry and transfer-layer formation rather than acting as an effective solid lubricant controlling friction magnitude [55,58].

### 3.4. Wear-Track Analysis—Optical Microscopy

**Figure 3:**
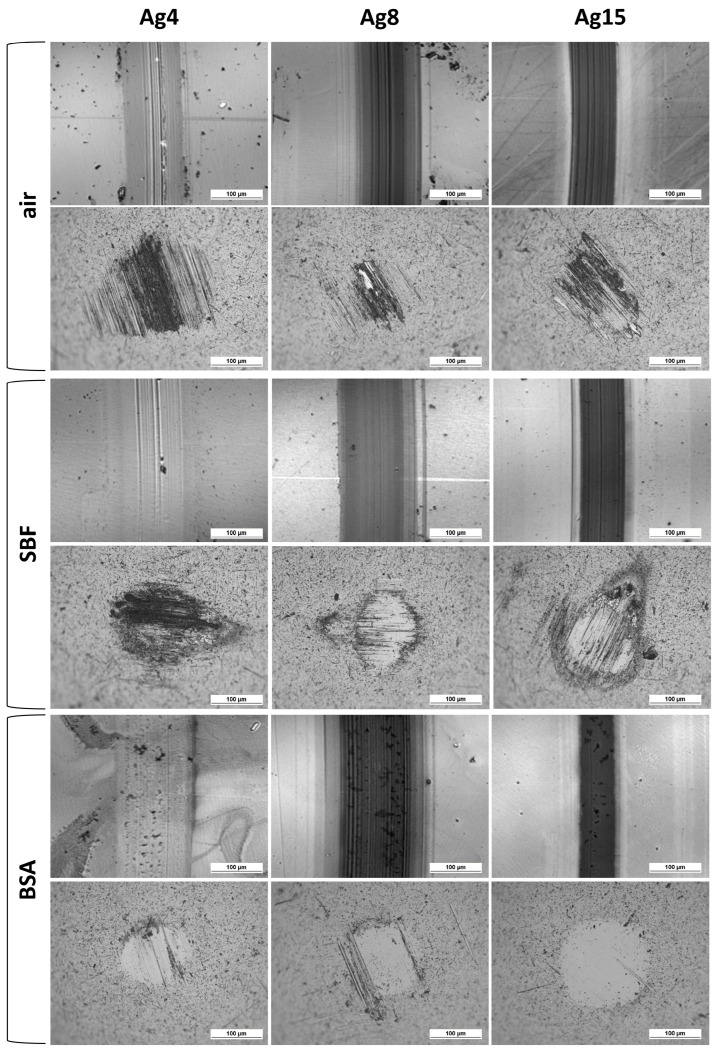
Optical microscopy images of representative wear tracks on Ag-DLC coatings and corresponding wear scars on steel counterparts after pin-on-disk tests performed in air, SBF, and BSA. Scale bar: 100 µm.

Optical microscopy was employed to qualitatively evaluate the morphology of the wear tracks formed on Ag-doped DLC coatings and the corresponding wear scars on the steel counterparts after tribological tests conducted in air, SBF and BSA environments. Representative optical images of the wear tracks and mating balls are shown in Figure 3.

For all investigated coatings, clear differences in wear-track morphology were observed depending on the testing environment. In air, the wear tracks are generally wider and more pronounced, exhibiting sharp boundaries and well-defined edges. The wear tracks formed under ambient conditions appear relatively uniform along the sliding path, which is consistent with the higher wear rates determined for this environment. These features are consistent with the more severe wear under dry sliding conditions and may reflect the contribution of abrasive and adhesive interactions.

In simulated body fluid (SBF), the wear tracks are visibly shallower and narrower than those formed in air. The track edges appear smoother, and the contrast between the wear track and the unworn surface is reduced. This observation suggests a milder wear regime, likely associated with the lubricating action of the aqueous medium and reduced direct solid–solid interaction during sliding. These qualitative observations are in good agreement with the lower wear rates obtained from profilometric measurements.

The most pronounced changes in wear-track morphology are observed for samples tested in the BSA environment. In this case, wear tracks are generally less distinct, with blurred boundaries and locally non-uniform appearance. In some regions, the wear tracks appear partially covered by tribofilms or transferred material, indicating the formation of a surface layer during sliding. Such features are consistent with the formation of a surface layer involving protein-derived material and/or wear debris, which may modify local contact conditions during sliding.

Quantitative values of representative wear-track depths measured on the coated disks are summarized in Table 3. These values provide a qualitative comparison of wear severity between different environments rather than absolute measures of wear volume. The highest wear track depths are observed for tests conducted in air, which corresponds well with the highest coating wear rates determined for this environment. Conversely, the smallest wear-track depths for coatings with higher silver content (Ag8 and Ag15) are recorded for tests in SBF, which is consistent with the wear rate results.

**Figure 4:**
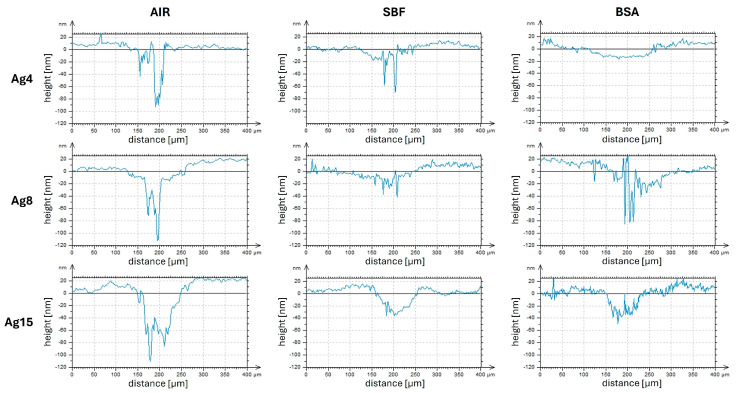
Representative cross-sectional profiles of the wear tracks after pin-on-disk testing for the investigated coating conditions. To ensure direct comparability, all profiles are presented using identical axis ranges (x-axis: 400 µm; y-axis: 145 nm). The profiles provide a qualitative visualization of the wear-track geometry and complement the wear-depth values reported in Table 3.

To complement the quantitative wear-track depth data summarized in Table 3, representative cross-sectional profiles of the wear tracks are presented in Figure 4. All profiles were plotted using identical axis ranges, which enables direct visual comparison of wear-track geometry under the investigated conditions. In addition to the numerical depth values summarized in Table 3, the profiles illustrate differences in the overall shape and severity of the wear scars. In general, the profiles obtained after testing in air exhibit more pronounced depressions, whereas those recorded in lubricated media are shallower, indicating a milder wear regime. At the same time, the BSA-tested samples show a more condition-dependent response, which is consistent with the complex interfacial processes occurring in the protein-containing medium. Therefore, the representative profiles should be regarded as a qualitative supplement to the quantitative wear data, providing additional insight into wear-track morphology under the investigated conditions.

Overall, optical microscopy observations qualitatively support the trends obtained from quantitative wear measurements; however, the image-based analysis should be interpreted as supportive rather than definitive evidence of the dominant wear mechanisms.

### 3.5. Wear-Track Analysis—SEM and EDS

**Figure 5:**
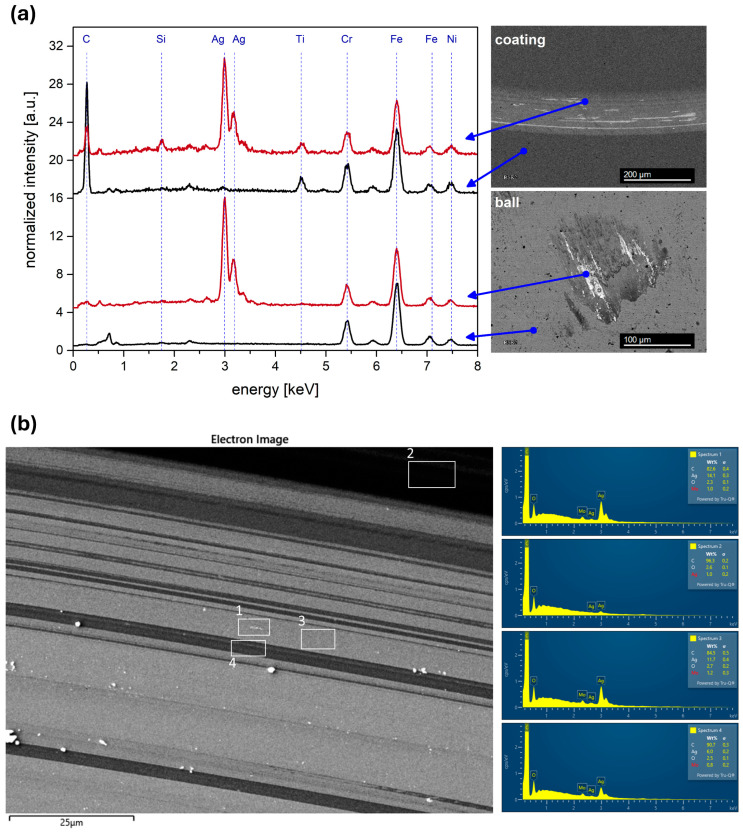
SEM micrographs and qualitative EDS analysis of the wear track formed on the Ag8 coating and the corresponding AISI 316L steel counterpart after pin-on-disk testing in air. (**a**) SEM images of the wear track (scale bar: 200 µm) and counterpart surface (scale bar: 100 µm), accompanied by qualitative EDS spectra collected from regions inside and outside the wear track in order to evaluate Ag redistribution and transfer of coating-derived material. The EDS intensity was normalized with respect to the Fe peak (~7 keV), enabling comparison of the relative Ag peak intensities. (**b**) SEM image of selected regions on the worn coating surface together with the corresponding EDS spectra.

In Figure 5a, the SEM images and EDS analysis of the wear tracks in the coating and counterpart for the selected Ag-doped DLC sample (Ag8) subjected to pin-on-disk tests in air are presented. It should be emphasized that the steady-state friction coefficient reflects the tribological response of the contact averaged over the entire sliding path and is largely governed by the properties of the deeper coating regions, where the contribution of silver is limited and the DLC matrix dominates.

In contrast, SEM/EDS analysis provides insight into localized phenomena within the wear track, such as the presence and redistribution of silver-rich conglomerates, which primarily influence the formation of the transfer layer rather than the macroscopic friction coefficient. The increased Ag signal detected inside the wear track and on the counterpart surface is consistent with the local accumulation and transfer of Ag-containing wear products. These observations suggest local transfer of coating-derived material; however, the present EDS analysis does not by itself demonstrate a continuous transfer film or its direct contribution to the macroscopic friction coefficient. In ambient conditions, friction-induced local structural modification of the near-surface DLC region may occur during sliding [59]. In the present study, this process should be regarded as one of the possible surface phenomena accompanying dry sliding rather than as the sole factor governing the overall friction response of the coatings. SEM and EDS examination of the wear scars also revealed the transfer of material from the Ag-doped DLC surface to the mating steel ball. The transfer film appears to be partially covering the Hertzian contact area.

As reported in our recent work [43], silver in Ag-incorporated carbon coatings occurs in two forms—dissolved in the amorphous carbon matrix and in conglomerates; these are randomly distributed on the whole surface. The qualitative EDS analysis revealed noticeable amounts of silver in both, inside the wear tracks (compared to the outside area) and on the surface of the counterpart. This is mainly caused by rubbing of the silver conglomerates and subsequent spreading of the worn material in the wear track and its transfer to the surface of the counterpart.

The qualitative EDS analysis (Figure 5b) suggested differences in the local chemical composition between the unworn surface and selected regions within the wear track. Area 2, representing the sample surface outside the wear track, was strongly dominated by carbon (96.3 wt%) and showed only a trace Ag contribution (1.0 wt%), which may indicate that the surface outside the main tribologically affected zone remained predominantly carbonaceous. In contrast, the regions analyzed within the wear track showed higher Ag contents. Area 1 exhibited the highest Ag contribution (14.1 wt%), which may be associated with locally smeared silver formed during the pin-on-disk test. Area 3, located within the wear track but outside the visibly smeared Ag-rich region, also showed a distinct Ag signal (11.7 wt%), which may suggest that silver remained present in the tribologically modified surface even in areas where no clearly visible Ag-rich particles were observed. Area 4, likely corresponding to a slightly deeper region within the wear track, exhibited an intermediate Ag content (6.0 wt%), which may reflect local variation in silver redistribution or retention within the worn layer. All analyzed regions remained carbon-rich, while the low oxygen content recorded in all areas may be related to slight surface oxidation or the presence of a thin tribochemically modified surface layer. Trace Mo detected in areas 1, 3, and 4 may be associated with local material transfer or the presence of wear debris within the contact zone.

### 3.6. AFM Analysis

**Figure 6:**
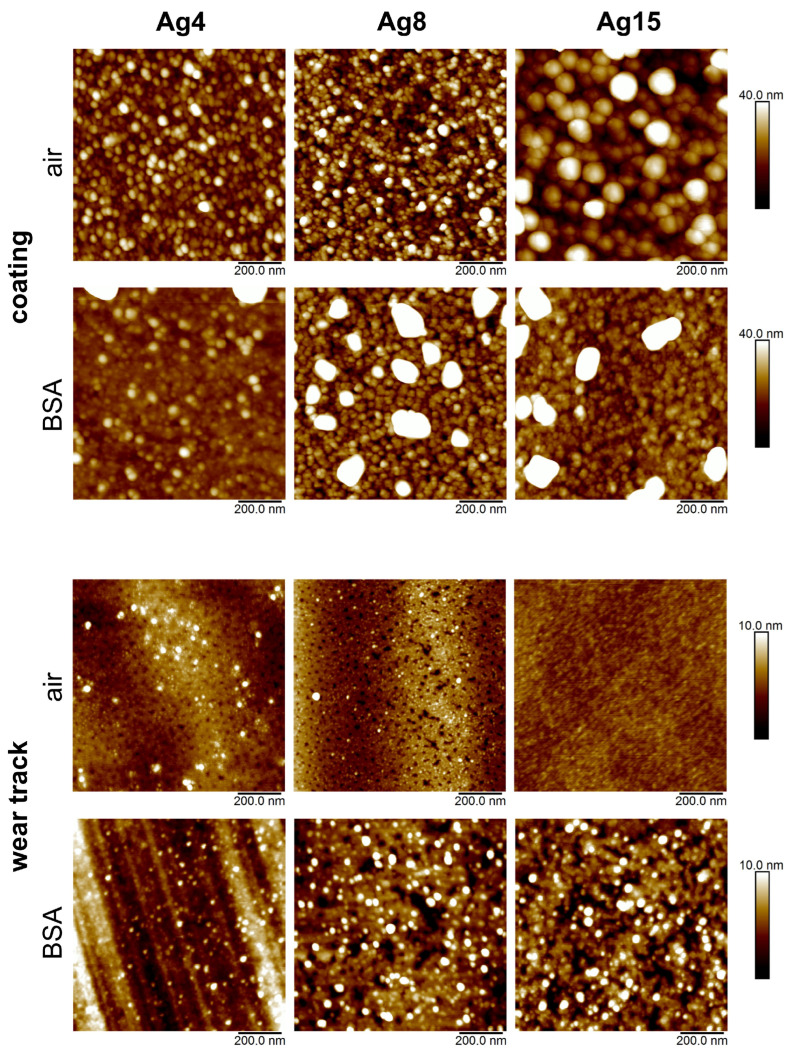
AFM topography images of Ag-DLC coatings acquired outside and inside the wear tracks after tribological tests performed in air and BSA. Scan size: 1 × 1 µm^2^.

Atomic force microscopy was employed to investigate the surface topography of Ag-doped DLC coatings before and after tribological testing, with particular emphasis on samples tested in the BSA environment. Representative AFM images acquired outside and inside the wear tracks are presented in Figure 6.

The as-deposited coatings exhibit a relatively smooth surface morphology for the Ag4 sample, whereas increasing silver content (Ag8 and Ag15) leads to a pronounced increase in surface roughness. This effect can be attributed to the formation of silver-rich globular domains distributed over the coating surface, which is consistent with previous observations reported for Ag-modified DLC coatings [59].

After tribological tests performed in the BSA environment, distinct changes in surface morphology are observed, especially within the wear tracks. Quantitative analysis of AFM images indicates that the BSA surface coverage parameter (SC) for coatings with higher silver content (Ag8 and Ag15) is more than twice that determined for the Ag4 coating, both before and after friction (Table 4). These results suggest that silver incorporation affects the formation of protein-related surface features, possibly through the combined effects of increased roughness, chemical heterogeneity, and interactions between adsorbed protein and tribologically generated debris.

A comparison of regions outside and inside the wear tracks reveals an approximately twofold reduction in the apparent BSA surface coverage after friction within the wear tracks. At the same time, AFM analysis shows a significant increase in the average size of surface agglomerates inside the wear tracks, with agglomerate heights being approximately two times larger than those observed outside the wear tracks.

Previous studies have demonstrated that protein adsorption inside wear tracks differs markedly from that on unworn surfaces due to tribologically induced denaturation and interaction with wear debris. These processes often result in the formation of large protein debris agglomerates that increase apparent surface roughness but do not represent continuous or intact protein layers [60,61,62].

It should be emphasized that the BSA surface coverage determined after tribological testing does not solely represent the amount of adsorbed protein. In addition to protein agglomerates, the calculated SC values may include contributions from wear debris generated during sliding. Such debris can act as nucleation sites for protein adsorption, become embedded within protein clusters, or promote their coalescence. Consequently, the actual amount of protein adsorbed on the surface after friction may be lower than indicated by the apparent surface coverage values determined from AFM analysis. AFM-derived SC should therefore be interpreted as a tribologically evolved composite layer rather than a pure protein film.

The increase in agglomerate size within the wear tracks is likely associated with the combined effects of protein–protein interactions, interaction between albumin and wear debris, and local changes in surface chemistry and topography induced by the sliding process.

The present results support the view that tribological interaction influences the evolution of protein-related surface structures within the wear track, although the exact contributions of adsorbed protein, denatured protein, and wear debris cannot be fully separated on the basis of the present AFM analysis alone [60].

### 3.7. FTIR Analysis

**Figure 7:**
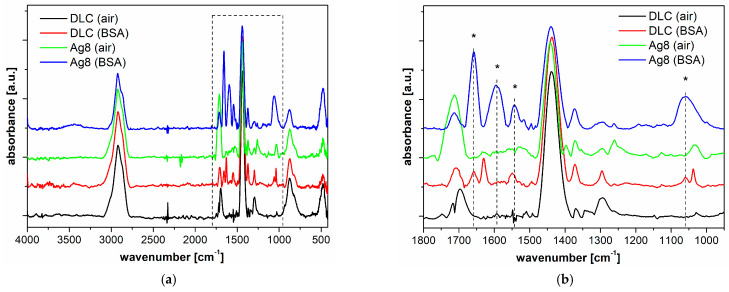
FTIR spectra of selected DLC and Ag8 coatings after tribological tests in air and BSA, measured in (**a**) the full wavenumber range and (**b**) a narrowed spectral range (dashed box from (**a**)) highlighting characteristic absorption bands. * Albumin functional group bands.

The FTIR spectra measured in the range of 4000–400 cm^−1^ for the two selected DLC and Ag8 coatings after tests in air and BSA environments are shown in Figure 7a. In order to visualize significant differences in the spectra, a narrower range of 1800–950 cm^−1^ was specified, which is shown in Figure 7b. In the spectra of DLC and Ag8 coatings tested in air, peaks originating from bonds typical for carbon coatings produced from methane (methyl, ethyl groups and carbon–oxygen bonds) are visible. The following peaks were distinguished in these spectra: in the range of 2990–2850 cm^−1^, the peaks originate from asymmetrical and symmetric vibrations stretching C-H bonds belonging to CH_3_ and CH_2_ groups; in the range of 1671–1750 cm^−1^, there is a peak coming from C=O stretching vibrations; in the range of 1470–1400 cm^−1^, there are peaks coming from CH_2_ scissor vibrations and asymmetric deformational CH_3_ groups; at 1370 cm^−1^, there are deformation vibrations of the C-H bonds belonging to the CH_3_ group; at 1294 cm^−1^, there are asymmetric vibrations stretching the C-O-C bonds, and at 479 cm^−1^, there is, in turn, a peak belonging to the bend vibration of C-O-C binding; at 1035 cm^−1^, there is a peak resulting from the vibrations stretching the C-O bonds in the C-OH group [63].

After the application of albumin on the examined coating surfaces, the course of the spectra changes slightly. The most significant changes were observed for the silver-doped DLC coating. In this spectrum, five characteristic bands assignable to albumin-related functional groups can be distinguished, which is consistent with the presence of protein-related species on the coating surface. However, FTIR alone does not provide definitive proof of the adsorption state, layer continuity, or the exact amount of adsorbed protein, and the spectroscopic observations should therefore be interpreted together with the AFM results and within the qualitative scope of the present study. In this case, the following bands have been separated: a wide band in the range of 3320–3520 cm^−1^, originating from asymmetric stretching vibrations found in primary amides; a wide band in the range of 1680–1620 cm^−1^, derived from primary amides belonging to the vibrations of C=O stretching and deformation N-H bonds; in the range of 1590–1580 cm^−1^, there are peaks originating from the N-H bonds of the primary and secondary amides; at 1557–1520 cm^−1^, there is a peak belonging to N-H bending vibration and also C-N stretching vibration. The last peak seen in the protein-derived spectrum is at 1058 cm^−1^, and it belongs to the C-N stretching vibrations found in the primary amides [64,65].

In the DLC coating spectrum, part of the peaks belonging to the protein and described above does not occur, and those that have appeared have low intensities compared to the Ag-doped coating. In this case, three weak bands were isolated at 1680–1620 cm^−1^, 1054 cm^−1^ and 1561–1532 cm^−1^. The latter vibration-related N-H bond is shifted relative to the band in the Ag-DLC coating spectrum. This indicates that the chemical environment has changed. The weaker protein-related bands observed for the undoped DLC surface may indicate a lower amount of protein-related material or a different interfacial chemical environment compared with the Ag-doped coating; however, this interpretation remains qualitative.

## 4. Conclusions

This study presents a tribological evaluation of Ag-doped DLC coatings tested in air and in physiologically relevant environments, including simulated body fluid (SBF) and bovine serum albumin (BSA). The results demonstrate that both silver incorporation and the testing environment significantly affect friction behavior, wear response, and surface interactions.

Increasing silver content leads to a systematic decrease in the H^3^/E^2^ ratio and, consequently, to increased coating wear compared to low-Ag DLC, regardless of the environment. In this system, H^3^/E^2^ appears to be an important parameter related to wear resistance, although the tribological response is also shaped by environmental and interfacial effects occurring during sliding.

Friction behavior shows a strong dependence on environment. Under dry sliding in air, all coatings exhibit similar steady-state friction governed primarily by the DLC-rich bulk. Among the tested media, the lowest steady-state CoF values were observed in SBF, indicating that the aqueous ionic environment provided the most favorable friction conditions. In the BSA environment, friction also remained low, suggesting that protein-related interfacial processes modified the contact conditions, although the CoF was not reduced below that measured in SBF.

Wear analysis indicates the highest coating wear in air and generally the lowest wear in SBF. Tests in the BSA environment result in less distinct wear tracks and evidence of surface layer formation, suggesting protein-mediated transfer processes. Wear of the steel counterparts remains negligible across all tested environments; however, due to the extremely low values, differences between conditions should be interpreted cautiously. The lack of a direct one-to-one relationship between steady-state friction and coating wear is observed in the present system; however, this should be regarded as an empirical tendency rather than a quantitatively established decoupling law.

Surface analyses by optical microscopy, SEM/EDS, AFM, and FTIR provide complementary but mainly qualitative insight into the tribological response of the investigated coatings. The results suggest silver redistribution, transfer of coating-derived material, and the formation of protein-related surface structures in the wear tracks.

Overall, the present study shows that Ag-doped DLC coatings exhibit environment-dependent friction and wear behavior under physiologically relevant test conditions. These findings are relevant for the design of multifunctional carbon-based coatings; however, the present work should be regarded as a tribological study rather than a full biomedical or antibacterial validation [65]. Future work should combine tribological testing with antibacterial, cytocompatibility, and long-term interfacial analyses in order to assess the practical applicability of Ag-doped DLC coatings in biomedical environments.

## Figures and Tables

**Table 1:** Chemical composition of the simulated body fluid (SBF) used in the tribological tests, prepared without TRIS buffer [49].

Reagent	NaCl	NaHCO_3_	KCl	K_2_HPO_4_3H_2_O	MgCl_2_6H_2_O	CaCl_2_	Na_2_SO_4_
Concentration (g/L)	7.996	0.350	0.224	0.228	0.305	0.278	0.071

**Table 2:** Plasticity index (H^3^/E^2^) of DLC and Ag-DLC coatings with different Ag contents, calculated from hardness and Young’s modulus values reported previously [43].

Sample	DLC	Ag4	Ag8	Ag15
Plasticity index [GPa] ^1^	0.131 ± 0.021	0.113 ± 0.023	0.106 ± 0.028	0.077 ± 0.017

^1^ Values are given as mean ± root mean square uncertainty.

**Table 3:** Disk wear-track depths measured for Ag-DLC coatings after pin-on-disk tests performed in air, SBF, and BSA. Values are given as mean ± standard deviation and are used for qualitative comparison of wear severity.

Sample	Disk Wear-Track Depth [nm]		
	Air	SBF	BSA
Ag4	96.4 ± 4.7	70.4 ± 6.8	28.3 ± 5.3
Ag8	127.0 ± 15.7	33.5 ± 8.0	98.7 ± 23.0
Ag15	112.8 ± 8.8	47.8 ± 5.0	54.2 ± 9.8

**Table 4:** BSA surface coverage (SC) and average agglomerate size determined from AFM images (scan size 10 × 10 µm^2^) of Ag-DLC coatings outside and inside the wear tracks after tribological tests in BSA.

Sample	BSA Surface Coverage (SC) [%]		BSA Average Agglomerate Size ^1^ [nm]	
	Coating	Wear Track	Coating	Wear Track
Ag4	5.95	1.85	29.3 ± 1.2	62.9 ± 6.5
Ag8	12.06	4.87	27.5 ± 0.3	87.3 ± 7.4
Ag15	11.39	5.62	31.9 ± 0.5	57.5 ± 1.6

^1^ agglomerate height.

## Data Availability

The original contributions presented in this study are included in the article. Further inquiries can be directed to the corresponding author.
